# Exploring bacterial cellulose as an engineered living and programmable biomaterial across disciplines through qualitative thematic analysis

**DOI:** 10.1038/s41598-025-01931-1

**Published:** 2025-05-23

**Authors:** Luis Quijano, Dagmar Fischer, Tiziana Ferrero-Regis, Laura Navone

**Affiliations:** 1 Faculty of Creative Industries, Education and Social Justice, Queensland University of Technology (QUT), Brisbane, QLD 4059 Australia; 2 Faculty of Science, Queensland University of Technology (QUT), Brisbane, QLD 4000 Australia; 3 ARC Centre of Excellence in Synthetic Biology, Queensland University of Technology (QUT), Brisbane, QLD 4000 Australia; 4 Division of Pharmaceutical Technology and Biopharmacy, Friedrich-Alexander-Universität Erlangen-Nürnberg, Cauerstraße 4, 91058 Erlangen, Germany; 5 FAU NeW-Research Center for New Bioactive Compounds, Nikolaus-Fiebiger-Straße 10, 91058 Erlangen, Germany

**Keywords:** Bioinspired materials, Bioinspired materials

## Abstract

Bacterial cellulose is an engineered living material which holds significant potential due to its material properties and broad applicability across scientific and design disciplines. However, challenges in interdisciplinary collaboration, scalability and commercialization have slowed its widespread adoption and integration into industry applications such as fashion and textiles. This study addresses the gap in understanding how bacterial cellulose is perceived, developed, and utilized across scientific and design disciplines. Through 20 semi-structured interviews with scientists and designers around the world, this paper explores the following themes: (1) the human-living material relationship spectrum, which highlights the distinct ways science and design stakeholders interact with bacterial cellulose as a living material; (2) perceptions of a living material made from bacteria; and (3) bacterial cellulose’s potential as a programmable biomaterial. Additionally, we employ Bruno Latour’s Actor-Network Theory to map the complex network of human and non-human actors shaping bacterial cellulose’s trajectory, identifying critical factors such as consumer acceptance, interdisciplinary collaboration, and material culture. By bridging perspectives from science and design, this study offers actionable insights into bacterial cellulose’s future as a sustainable and programmable engineered living material, guiding its responsible development and broader adoption across industries.

## Introduction

At the forefront of sustainable innovation, bioinspired materials such as bacterial cellulose are reshaping our approach to environmental challenges^1^. Bacterial cellulose, a versatile and biodegradable polymer produced by various bacterial species, stands out for its promising applications in a wide range of disciplines^2^ such as biomedicine and pharmaceutical sciences^3^, fashion and textiles^4^, cosmetics^5^ and food^6^. In fact, a recent bibliometric review identified 62 distinct academic fields spanning between the sciences and social sciences that intersect with bacterial cellulose research^7^. This diverse interest underscores the material’s prominence as a key biomaterial in the nexus of emerging materials, signaling a shift towards more eco-conscious and resource-efficient practices.

Bacterial cellulose is produced primarily by *Komagataeibacter xylinus* and is distinct from plant-derived cellulose due to its inherent advantages such as higher purity, mechanical strength, and biocompatibility. Bacterial cellulose can be cultivated under diverse conditions including static, agitated, and bioreactor fermentation, as well as variations in temperature, pH, and nutrient sources^2,8^. Notably, it has demonstrated a remarkable ability to grow with a myriad of substrates and feedstocks, many of which are by-products or waste materials^8^. This adaptability positions bacterial cellulose at the forefront of sustainable biomaterials, suggesting a pivotal role in addressing environmental challenges, reducing reliance on virgin resources, and fostering circular economy practices.

While bacterial cellulose gains recognition as an engineered living material, the concept of sustainable biomaterials is evolving to encompass materials that are not only derived from nature but also possess dynamic and programmable functionalities. Engineered living materials represent a cutting-edge approach where either living organisms are integrated into materials to confer specific functionalities or properties or the materials themselves are instilled with such attributes^9^. Engineered living materials like bacterial cellulose, not only serve a functional purpose but exhibit dynamic, responsive, and more-than-human characteristics^10–12^. Table 1 highlights key material properties of bacterial cellulose, showcasing its functional potential for modifications across diverse applications.

**Table 1:** Material properties of bacterial cellulose.

Category	Property	Description	Characterization
Biological characteristics	Biocompatibility	Compatibility with human tissues^2^	In vitro: No cytotoxicity observed in Swiss 3T3 fibroblasts and INS-1832/13 rat insulinoma cells^13^ In vivo: Mild inflammation at 1-week post-implantation, diminishing to minimal inflammation by 2 weeks, provided proper endotoxin clearance is achieved^13^
	Biodegradability	Naturally degrades under biological conditions^3^	BC polymers: ~ 75% mass loss after 8 weeks of soil burial^14^ BC films: ~81% mass loss after 150 days of soil burial^15^
	Purity	Free of lignin, hemicellulose and other plant impurities^16^	~ 99% pure cellulose content^16^
Mechanical characteristics	Tensile strength	High mechanical stability; stronger than plant cellulose in wet conditions^17^	Range: 5–954 MPa, depending on production methods and treatments^18–20^ Typical Values: 70–300 MPa for unmodified BC films^18^; up to 437 MPa with titanium cross-linking^19^; 758 MPa achieved in ultrathin films^20^
	Elastic modulus	Young’s modulus	Unmodified BC films: 5–17 GPa^18^ Single BC filament: 114 GPa^21^ Single BC fiber: 78 ± 17 GPa^22^
Physical characteristics	Water Content	High water retention, making it highly absorbent^3^	> 90%^3^
	Porosity	Customizable pore structure for different applications^23^	Up to 94% in fully hydrated BC membranes, based on water content and swelling ratio analysis^24^
	Transparency	Can be modified to be highly transparent^25^	Up to ~ 89%^25^

In this paper, *alive* and *living* refers not only to potential living bacteria components within the material that enable it to respond to environmental stimuli, but also to the material itself which possesses *living* attributes. This living intersection between biology and materials science opens new possibilities for creating materials with unprecedented properties, such as self-healing capabilities, adaptability, and environmental responsiveness^26–28^. *Adaptable* highlights its ability to be engineered or modified for specific applications in a wide array of fields such as synthetic biology, material science, pharmacy and fashion. *Programmable* refers to the precise control and direction of the material’s behavior or properties through specific inputs or genetic modifications, allowing for tailored functionalities^29^. The term *more-than-human* stems from the social sciences and refers to the recognition that these materials transcend human-centered design, incorporating and interacting with biological systems in a way that challenges traditional boundaries^30^. This more-than-human perspective invites us to rethink our relationship with materials, considering them as active participants in a broader ecological context.

This convergence of living materials and material design also presents a paradigm shift in how we conceive and utilize materials, paving the way for a more sustainable and ecologically harmonious future^31^. Material design, in the context of a circular economy, focuses on creating products and systems that are sustainable, environmentally friendly, and contributes to closing resource loops^32,33^. Yet, the incorporation of engineered living materials into the fabric of our daily lives raises profound socio-cultural implications, necessitating an exploration beyond laboratory modifications^34^. Understanding how stakeholders perceive and interact with bacterial cellulose as a case study becomes integral in navigating the potential socio-cultural impact of these materials. This study provides the first comprehensive analysis of how scientists and designers across diverse disciplines worldwide interact with bacterial cellulose, uncovering the varied ways in which it is understood, applied, and incorporated into their respective fields. This paper seeks to illuminate the emerging roles and multifaceted identity of bacterial cellulose, as uncovered through the unique lens of stakeholder perspectives. By conducting a series of semi-structured interviews, this study aims to answer three critical research questions:

1. What stakeholders engage with bacterial cellulose, and why?
2. What is the future role and identity of bacterial cellulose as a living and programmable biomaterial?
3. What socio-cultural implications and challenges do stakeholders foresee in the integration of bacterial cellulose as an engineered living material into industrial applications and daily life?

## Methodology

Bacterial cellulose was chosen for this study due to its prominence as a well-studied engineered living material that has demonstrated commercial scalability and multidisciplinary significance. Unlike other engineered living materials that may remain confined to laboratory research, bacterial cellulose has been successfully developed at both industrial and artisanal levels, making it a unique case study for understanding material adoption across different contexts. Bacterial cellulose has been investigated across 62 academic disciplines, ranging from material science and biotechnology to design and social sciences, illustrating its broad applicability and research interest^7^. Furthermore, bacterial cellulose’s ability to be grown in decentralized settings—such as home laboratories, artisanal workshops, and do-it-yourself biofabrication spaces—sets it apart from other biomaterials that typically require sophisticated laboratory environments^4^. Given its dual presence in scientific and design communities, bacterial cellulose offers a valuable lens through which to explore multidisciplinary material development, stakeholder engagement, and the broader socio-cultural implications of integrating engineered living materials into practical applications.

This study delves into the diverse motivations and perceptions shaping the engagement of various stakeholders, including scientists and designers ranging from industry to academia with bacterial cellulose. Given the exploratory nature of this research, a qualitative method (semi-structured interviews) was employed to gather a comprehensive understanding of bacterial cellulose’s emerging identity. Qualitative methods are, “focus[ed] in understanding a research query as a humanistic or idealistic approach…. [and] used to understand people’s beliefs, experiences, attitudes, behaviour and interactions…”^35^. Semi-structured interviews allow for interviews to be focused yet flexible enough to explore relevant ideas that may come up during the interview^36^. In addition, semi-structured interviews are the preferred data collection method when researchers seek to understand participants’ perspectives rather than phenomena. For this paper, the authors follow the framework for semi-structured interviews outlined in Seven Steps to Conducting, Analyzing, and Reporting Semi-Structured Interview Data (7S CARS-SID)^36^. Currently, advancements in bacterial cellulose primarily emerge from scientific and laboratory experiments. As such, using qualitative methods can provide unique insights beyond what can be observed within the confines of a laboratory setting.

### Data collection

From February 2022 to February 2023, 20 semi-structured interviews were conducted, employing purposive sampling to ensure a diverse range of perspectives^37^. Before the interviews were collected, the study was approved by the university’s Human Research Ethics Committee (approval number 2000001098). All methods were performed in accordance with the relevant guidelines and regulations. Verbal informed consent was obtained before each interview and from the individual(s) for the publication of any potentially identifiable data included in this article. These interviews, each lasting between 30 and 60 min, were carried out via Zoom. The sample size was strategically chosen to provide rich, in-depth insights, informing future directions in bacterial cellulose research and its applications. Interviewees were selected based on their active involvement in bacterial cellulose-related fields, ensuring a varied yet relevant participant pool. Recruitment was conducted through targeted emailing, leveraging professional networks and industry contacts, and participants were informed that they could opt out of the interview at any time with no penalty.

The interview structure commenced with questions about each participant’s background, including their educational and professional experiences. This was followed by inquiries into their specific practices and experiences with bacterial cellulose, probing into the challenges and learning curves encountered. Discussions also extended to their experiences in interdisciplinary collaborations involving bacterial cellulose and participants’ perceptions of how they interact with bacterial cellulose and bacterial cellulose producers. In concluding the interviews, participants were invited to offer additional insights or thematic comments, enriching the data with spontaneous and unscripted reflections.

### Data analysis

With consent, all interviews were audio-recorded and transcribed verbatim. The transcribed data underwent analysis through Atlas.Ti software, following strict confidentiality and anonymity protocols. Identifying information was anonymized, and participants were assigned acronyms to ensure confidentiality. Using inductive thematic analysis, several prevalent themes were revealed, offering a nuanced understanding of bacterial cellulose’s role and perception among stakeholders. Initial themes, autonomously identified by the primary author, underwent a collaborative review and refinement process with the research team to ensure the analysis’ reliability and validity, following Bryman’s guidelines^38^. An open-coding process was first used to identify emergent themes, which were then iteratively refined through comparison across participant groups (scientists and designers). Coding outcomes and theme development were regularly discussed among the co-authors to ensure analytical rigor and reduce potential researcher bias.

To further enhance the trustworthiness of the analysis, we followed standard qualitative practices such as member checking, peer debriefing and data saturation to reduce bias analysis. Member checking allowed interview participants to confirm the authenticity of their accounts by reviewing and correcting their transcripts after the interviews were conducted. Peer debriefing among co-authors provided an additional layer of validation, particularly in refining the coding process and thematic structure. Data saturation—often reported in qualitative studies as occurring between 9 and 17 interviews^39^—was achieved by the 20th interview affirming the sufficiency of the sample. These measures collectively strengthened the robustness of the findings and ensured that interpretations presented in this exploratory study remained grounded in participant perspectives.

## Results

### Participant demographics

This study explores a broad array of perspectives on bacterial cellulose, drawing from a diverse group of professionals whose expertise spans across the fields of fashion, textile science, business, material science, and biotechnology amongst other scientific disciplines. The interview participants represent a global perspective, hailing from 11 countries across North America, Europe, Asia, and Australia, reflecting the wide-reaching interest and applications of bacterial cellulose. Table 2 on the next page features a detailed description of the 20 participants involved in the interviews. It is important to note that this wide range of stakeholders work with bacterial cellulose in a variety of different ways. Many participants are involved in laboratory research or have experience developing bacterial cellulose with scientific experimentation. Others from the participant group are also experienced in working with bacterial cellulose with artisanal methods or in business. Some participants focus on bacterial cellulose on a micro or nano scale, whereas other participants work with bacterial cellulose in a bigger material quantity. The following section will take these points into consideration and discuss bacterial cellulose as a living and programmable material from a systems and top-down approach.

**Table 2:** Description of participants.

Code	Background	Job title	Country
P1	Fashion (Business)	SME Owner	Canada
P2	Fashion (Business)	SME Owner	Sweden
P3	Fashion (Design)	Senior Lecturer	Australia
P4	Fashion (Business)	SME Owner	Australia
P5	Fashion (Creative Arts)	Practitioner	Austria
P6	Science (Textile Science)	Associate Professor	United States of America
P7	Fashion (Business)	Chief Operational Officer	United States of America
P8	Fashion (Business)	SME Owner	United States of America
P9	Science (Textile Science)	Associate Professor	India
P10	Fashion (Creative Arts)	Postdoctoral Research Fellow	United Kingdom
P11	Science (Synthetic Biology)	Professor	United Kingdom
P12	Science (Biotechnology)	Industry	Denmark
P13	Science (Textile Science)	Lecturer	United Kingdom
P14	Science (Microbiologist)	Chief Scientific Officer	Denmark
P15	Science (Chemical Engineering)	Postdoctoral Research Associate	Australia
P16	Science (Material Science)	Tenured Scientist	Spain
P17	Fashion (Creative Arts)	Practitioner	Australia
P18	Science (Synthetic Biology)	Postdoctoral Research Associate	Australia
P19	Fashion (Business)	Co-founder/Chief Executive Officer	Hungary
P20	Science (Biotechnology)	Assistant Researcher	Portugal

### Bacterial cellulose and its emerging material identity

In the realm of living materials, bacterial cellulose emerges as a pivotal player due to its unique properties and potential applications across diverse disciplines. This section delves into the intricate identity and material agency of bacterial cellulose, dissecting how it is perceived and utilized by various stakeholders in science, technology, design, and beyond. The themes within this paper include: (1) the human-material relationship spectrum of participants interacting with bacterial cellulose and its bacterial producers as a living material, the (2) perception of a living material made from bacteria, and (3) bacterial cellulose’s role as a programmable material.

#### Human-living material relationship spectrum

This subsection explores the human-living material relationship spectrum, ranging from pragmatic utilization to deep ecological integration. It sheds light on how the living nature of this biomaterial influences its acceptance, application, and innovation potential in various fields. At the core, living materials, such as bacterial cellulose, consist of dynamic, responsive, more-than-human elements^30^. This concept extends beyond the material itself to include the organisms that produce it and the relationships between humans and these life forms as ‘engineered living materials’ have their own material agency^40^. Our findings reveal a spectrum of relational attachments to these organisms, ranging from purely utilitarian to deeply empathetic and holistic approaches.

**Fig. 1:**
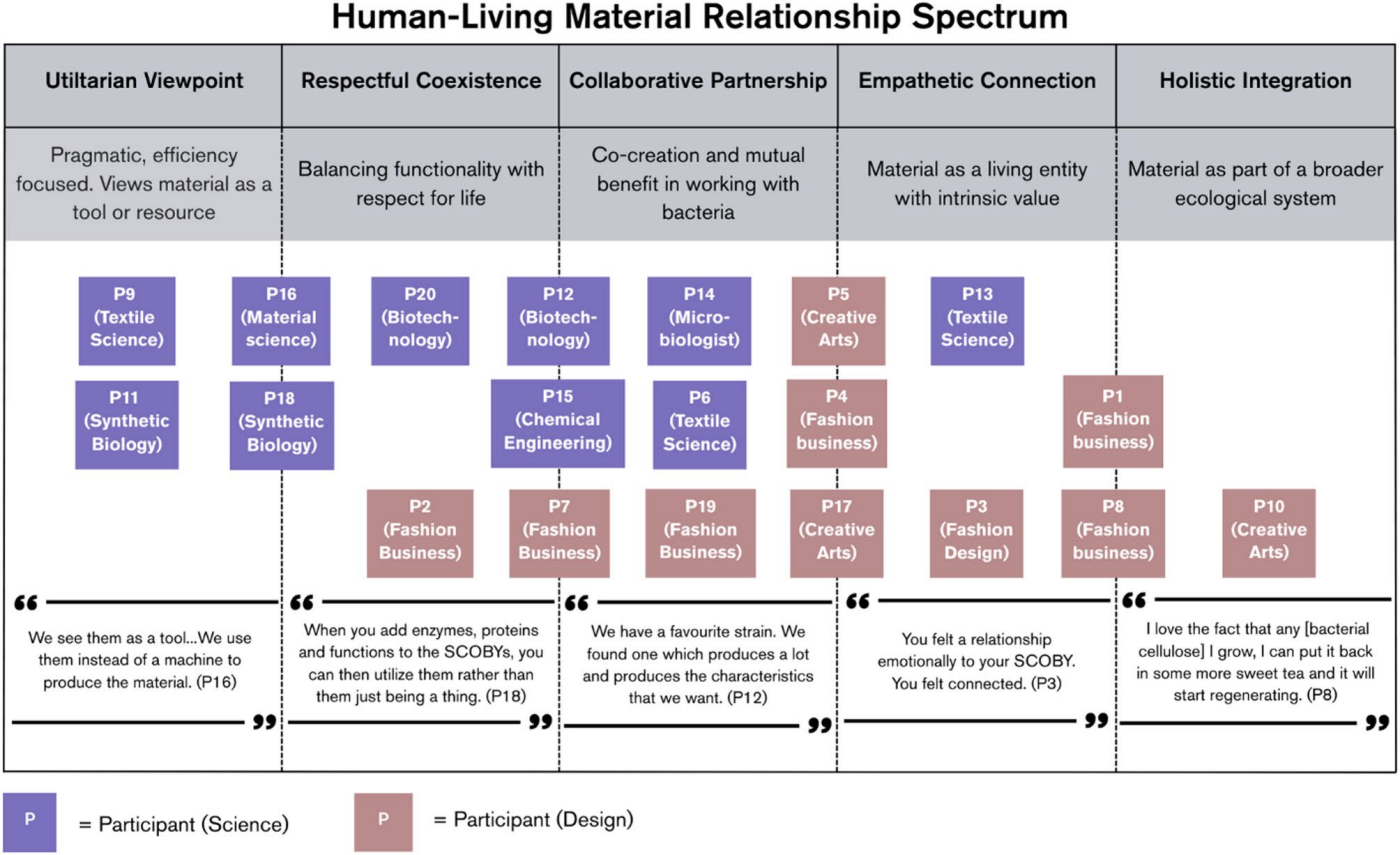
The human-living material relationship spectrum visually depicts the diverse ways stakeholders—scientists and designers—perceive and engage with bacterial cellulose. The categories progress from viewing the material as a functional tool (utilitarian viewpoint) to seeing it as an interconnected entity within a broader ecological system (holistic integration). Participants in blue squares are from the scientific community whereas participants in red squares come from the design field.

To visually depict this spectrum of differing relationships, Fig. 1 on the next page shows the human-living material relationship spectrum. As seen in the figure, the blue squares represent participants from the scientific community, while the red squares denote participants from the design field. This color-coding reflects the varying disciplinary backgrounds of the stakeholders, each contributing unique perspectives to their engagement with bacterial cellulose. In addition, the dotted lines between the spectrum categories highlight the fluidity of these relational viewpoints. Stakeholders may overlap between categories as their perspectives and engagements evolve over time. These overlaps reflect the complexity of human-material interactions, where perceptions and practices can be context-dependent and responsive to evolving experiences. Table 3 on the next page extends upon the categories within the human-living material relationship spectrum.

Participants were categorized based on how they discussed their interactions with bacterial cellulose and its microbial producers during the interviews. These interactions were shaped by their specific roles, experiences and goals, revealing how they perceive the material and its potential in their respective fields. The categories that emerge from these conversations reflect the varying degrees of attachment and integration, from seeing the material as a functional tool to recognizing it as a living entity with intrinsic value. This categorization allows us to better understand the diverse ways in which bacterial cellulose is viewed and utilized across disciplines, helping us appreciate the material’s potential in a wide range of applications.

**Table 3:** Human-living material relationship spectrum categories.

Category	Key characteristics	Approach to bacterial cellulose	Interaction style	Stakeholder perspectives
Utilitarian viewpoint	Pragmatic, efficiency focused. Views material as a tool or resource	Goal-oriented, emphasizing tangible results and productivity	Objective and outcome driven. Focus on efficiency and functionality	Concerned primarily with results and benefits derived from bacterial cellulose
Respectful coexistence	Recognizes living nature but maintains a functional relationship. Seeks a balance between utility and respect for the living entity	Respect for living nature coupled with a focus on functional outcomes	Pragmatic yet considerate. Appreciation of biological properties alongside functional outcomes	Appreciates biological properties but interaction remains grounded in achieving specific outcomes
Collaborative partnership	Views bacterial cellulose as a collaborative partner; mutual benefit and co-creation process	Balanced engagement, acknowledging mutual benefits and contributions	Interactive, cooperative. Nurturing and growing the microbes and materials	Engages with bacterial cellulose in nurturing and fostering growth, considering the responsiveness of the material
Empathetic connection	Deep personal connection, attributing human-like qualities. Sees it as a living being with intrinsic value	Views bacterial cellulose as a living being, fostering a deeper connection	Emotionally engaged and personal. Caring and perceiving material as part of creative expression	Engages with bacterial cellulose on a personal level, often confronted by its smell, touch, and flesh-like appearance
Holistic integration	Profound understanding and acceptance. Views material as part of a larger ecosystem	Holistic approach, caring for material as a living entity within an ecological context	Ethically driven, responsible stewardship. Considering broader ecological impacts	Emphasizes responsible stewardship and ethical considerations, viewing bacterial cellulose as intricately woven into the broader ecosystem

This table outlines the distinct aspects of each category in the human-living material relationship spectrum, providing a clear comparison of how stakeholders’ perceptions and interactions with bacterial cellulose vary across the spectrum.

##### Utilitarian viewpoint (scientists only)

“Microbes to some extents are algorithms of evolution turned into little machines and that’s basically it,” stated Participant 11, Professor in synthetic biology in the United Kingdom. The utilitarian perspective reflects a pragmatic approach towards bacterial cellulose and the microbes producing bacterial cellulose, considering them primarily as functional tools or resources. Stakeholders adopting this viewpoint appreciate the material’s properties but emphasize its practical applications. For example, Participant 16, a material scientist in Spain describes bacterial cellulose and its microbial producers as a ‘means to an end’ stating, “We see them as a tool…We use them instead of a machine to produce the material.” This perspective prioritizes efficiency and functionality, with less concern for the microbes or the material beyond their practical roles.

Many participants highlighted the advantages of bacterial cellulose based on their specific fields of application, including its higher purity compared to plant cellulose, biodegradability, and sustainability. Furthermore, interactions with the bacteria and the material itself are often centered on optimizing production and enhancing material properties. When discussing potential improvements, Participant 16 elaborates on the need for increased efficiency in bacterial cellulose production, explaining, “If I could change one thing from bacterial cellulose, I would improve its efficiency so that the bacteria can grow faster, and they can produce more bacterial cellulose.” This sentiment reflects a broader trend among researchers focused on streamlining bacterial cellulose synthesis to meet industrial and commercial demands.

Several participants highlighted the importance of productivity in bacterial cellulose production, particularly in relation to strain selection and cultivation methods. Participant 18, a postdoctoral research associate in synthetic biology in Australia contrasted the efficiency of working with a single bacterial cellulose-producing strain versus a symbiotic colony of bacteria and yeast (SCOBY) derived from kombucha tea. Reflecting on the advantages of controlled optimization, they explained:

“Work[ing] with a single strain, you can optimize your industrial production … so you can get the optimal growth, temperature, growth, and weight. You cannot do that with the [SCOBY] community because you must consider all the organisms present which are somehow linked to the production, which means you must compromise on certain factors.” (Participant 18).

This perspective underscores a distinction between industrial-scale bacterial cellulose production and more artisanal or community-based approaches. While single-strain optimization enables greater control over environmental variables and yield, working with a microbial consortium introduces additional complexities, requiring researchers to balance interactions among multiple species.

Beyond the technical refinements sought in bacterial cellulose production, utilitarian viewpoint participants direct their attention primarily towards the end-product and how the material or microbes can help them achieve it. In this approach, stakeholders prioritize efficiency, scalability, and material performance, with less emphasis on the biological intricacies of the microbes involved. One of the reasons bacterial cellulose is approached in this way is its inherent consistency – a key advantage over other biomaterials. As Participant 9, Associate Professor in textile science in India expounds, “The best out of bacterial cellulose comparatively [to mycelium leather] is that we can see the consistency in [its] production.” For many scientists, modifying bacterial cellulose to achieve superior properties for targeted applications is central to their research. This includes not only enhancing the material itself but also engineering the microbes to maximize output and functionality.

In summary, utilitarian viewpoint stakeholders view bacterial cellulose less as a dynamic, living material and more as a malleable resource—one that can be tailored to meet specific industry needs. Ultimately, this approach prioritizes extraction efficiency, yield optimization, and the tangible advantages offered by the final product, reinforcing a results-driven outlook on microbial biomaterial production.

##### Respectful coexistence (mainly scientists, few designers)

In the realm of respectful coexistence, stakeholders acknowledge the living nature of bacterial cellulose while maintaining a functional relationship. This viewpoint is exemplified by Participant 15, a postdoctoral research associate in chemical engineering in Australia, who reflects on the dynamic interaction between the bacteria and the material in their research:

“I have been doing experiments on how the [bacterial cellulose] bacteria move and make their structures. You can see the tiny bacteria communicate and move… how they interact, how they make their structures. You are seeing the community of the bacteria and how they make something. This is one of the main reasons I like them to be honest.” – Participant 15.

There is a recognition and respect for the material’s intrinsic vitality, yet interactions remain primarily goal oriented. Participant 16, a material scientist in Spain mentions echoes this attitude when discussing the environmental impact of bacterial cellulose, stating, “We use bacteria that is more friendly for the environment.” This sustainable approach seeks a balance between utility and acknowledgement for the living entity. However, participant 20, an assistant researcher in Portugal, cautions, sustainability cannot be assumed based solely on the bioprocesses involved: “While the bioprocess [is a] more environmentally friendly approach, the chemical processes does not mean that it is green or environmentally safe.” There are extra considerations for sustainability to consider in addition to the material and microbes.

Participant 18, a postdoctoral research associate in Australia, further elaborates on the functional enhancement of bacterial cellulose, particularly when working with SCOBYs (Symbiotic Communities of Bacteria & Yeast). They note: “When you add enzymes, proteins and functions to the SCOBYs, you can then utilize them rather than them just being a thing.” This statement reflects an understanding of the living nature of microbes, coupled with a focus on enhancing their utility. Participant 12, a molecular biologist at a biomaterial company in Denmark offers further insight into the appeal of working with non-genetically modified (GMO) bacterial cellulose producers saying, “It’s been really nice that it’s just a non-GMO organism that we work with… We don’t have to have a classified lab or anything. We can throw away [lab waste] normally. That’s the big, huge advantage of it.” For this participant, working with non-GMO bacterial cellulose aligns with a philosophy of simplicity and environmental responsibility. The absence of GMO reduces the regulatory burden and allows for a more straightforward, sustainable approach to lab work.

In conclusion, participants who adopt the respectful coexistence viewpoint recognize bacterial cellulose and its material producers not just as biological curiosities but as dynamic components capable of contributing significantly to their work, be it in research, design, or industrial production. This approach is characterized acknowledging its living essence while channelling its potential towards tangible applications.

##### Collaborative partnership (mixed group of scientists and designers)

At the midpoint of the spectrum, collaborative partnership embodies a balanced approach where stakeholders perceive themselves as collaborators with bacterial cellulose producing strains and the material. There is mutual recognition of the co-creation process, where both human and microbial agents influence the outcome, fostering a relationship marked by respect for the bacteria and responsiveness from the material. Participant 17, a creative practitioner in Australia explains how they approach bacterial cellulose producers in their artisanal practice, emphasizing the careful, respectful relationship they maintain with the material:

“Within my practice, I’m always mindful of how I’m working with it, and I never deliberately kill it. The only times I’ve ever had it die is when I was working with [others] , and they didn’t follow the instructions. And just before Christmas, I lost 12 months’ worth of research to possums.” (Participant 17).

Here, the creative practitioner expresses a deep awareness of the bacterial cellulose’s life cycle, highlighting the intentionality and care involved in the process. The loss of material due to external factors underscores the challenges of working with living materials that require constant attention and respect.

Following up with an account from the scientific perspective, Participant 12, a molecular biologist working at a biomaterial company in Denmark reflects on the importance of selecting and nurturing specific bacterial strains that align with desired outcomes: “We have a favourite strain. We found one which produces a lot and produces the characteristics that we want.” In both design and scientific practice, collaborative partnership can take a significant amount of time and resources to achieve as the relationship with bacterial cellulose-producing strains is not merely transactional but built over time. In many cases, they must be modified or adapted to meet specific performance criteria, and these modifications can take years—or even decades—to achieve. These innovations, especially when pursued on an industrial scale, are often protected through intellectual property rights such as patents, further emphasizing the value placed on the material’s and microbe’s unique properties and the time invested in optimizing them.

When scaling the production or considering which strains to take towards commercialization as collaborative partners, there may be a need to revisit bacterial cellulose artisanal practices and re-examine what already occurs in traditional industries. Participant 15, a postdoctoral research associate in chemical engineering in Australia draws attention to the scalability of bacterial cellulose production, referencing traditional artisanal practices that have been successfully implemented in Southeast Asia: “I think we can scale the bacterial [cellulose] production as people in the Philippines, Thailand and Indonesia do with nata de coco.” Nata de coco, a form of bacterial cellulose in the jelly-like textures often used as toppings for Southeast Asian desserts and in drinks, demonstrates the potential to meet both local and international demand.

Overall, collaborative partnership serves as a bridge between the utilitarian viewpoints and respectful coexistence on the one hand, and empathetic connection and holistic integration on the other. At this juncture, there is a significant realization that the human influences the microbial design and that both play a role with each other in the co-creation process. This is stronger than mere acknowledgment; it involves a deeper, active recognition of how humans shape and are shaped by their microbial partners.

##### Empathetic connection (mainly designers, few scientists)

Moving further along the spectrum, some participants exhibit an empathetic connection that goes beyond a functional relationship and attributes pet-like qualities to bacterial cellulose. This stage is marked by a deep, personal connection where bacterial cellulose is viewed not just as a material but as a living being with its own intrinsic value. Participant 3, a Senior Lecturer in fashion design in Australia illustrates this relationship through a design project in their courses that incorporated kombucha SCOBY, noting, “You felt a relationship emotionally to your SCOBY. You felt connected.” This sentiment was echoed by student participants, who nurtured their SCOBYs by feeding them culture media and observing their growth. The emotional connection grew as the SCOBYs transformed into tangible design materials. This connection is not limited to the designer’s intention but also reflects a shared experience between the designer and the material itself. Further emphasizing this emotional connection, Participant 17, a creative practitioner in Australia describes the sensory aspects of working with bacterial cellulose: “[People] are often confronted by the smell, the visceral nature, the touch, the flesh-like [appearance of bacterial cellulose].” This sensory engagement goes beyond utility and demonstrates how the material can invoke visceral and emotional reactions in those who interact with it. The tactile and olfactory qualities of bacterial cellulose transform it from a simple biomaterial into something more personal and alive.

Participant 13, a textile scientist in the United Kingdom reinforces this notion of empathy by describing their nurturing relationship with the material, “I feed them, clean them, look after them, and keep them warm. I really have that emotional bond because I look after it and I know. . .all my work stems from [them].” Having worked with bacterial cellulose for years—first through kombucha SCOBYs and later in a laboratory setting—Participant 13 emphasizes the care involved in cultivating the material. They also illustrate the division of labor between their home environment and laboratory, “When I want to grow larger sheets, I grow it at home. When I want to grow smaller, tiny pieces for analysis, I grow it in the lab because I can control that easily.” In a similar vein, Participant 8, the CEO of a company producing bacterial cellulose-based clothing using artisanal methods in the United States, shares their own personal connection with the material, “I find myself speaking to the [SCOBY] cultures. . .I call them my mamas. There’s a sense of companionship and care that goes into nurturing them.” This statement underscores the deeply nurturing and almost familial relationship some individuals form with bacterial cellulose. The practice of speaking to and caring for the cultures reflects an emotional investment that transcends the material’s utilitarian function.

In summary, empathetic connection is evident in the way individuals interact with bacterial cellulose, often attributing personality, and life to the material and microbes. They engage in practices that reflect a nurturing and caring approach, acknowledging the material’s unique characteristics, and responding to them with a sense of wonder and respect. The empathetic connection stage represents a shift to recognizing bacterial cellulose as a partner in the creative process. It is a relationship where the material and microbes are not only used but also cherished, fostering a bond that is both personal and profound.

##### Holistic integration (designers only)

At the pinnacle of the human-living material relationship spectrum, holistic integration represents a profound and interconnected understanding of bacterial cellulose, transcending its materiality. Stakeholders subscribing to this perspective view bacterial cellulose not merely as a resource, but as a living entity intricately woven into the fabric of a broader ecosystem. This viewpoint demands a philosophy grounded in care and respect, akin to the principles governing interactions with any living organism. As Participant 8, CEO of a company producing bacterial cellulose clothing using artisanal methods in the United States shares, “They are alive, and I am very, very careful. I do not do anything that is toxic for the bacteria. I love the fact that any [bacterial cellulose] I grow, I can put it back in some more sweet tea and it will start regenerating, even a dress.” This perspective emphasizes a deep respect for the life cycle of bacterial cellulose, with a recognition that the material is part of a regenerative cycle. The idea that a material can regenerate—much like a living organism—speaks to an understanding of bacterial cellulose as not just a product to be consumed, but an entity capable of self-renewal and contributing to an ongoing cycle of life.

Participant 10, a postdoctoral research associate in creative practice in the United Kingdom expresses a similar sentiment in their artisanal studio work saying, “I would place notes under my materials as they do, to say, you are wanted, and you are loved. And I would watch them grow.” The practice of writing notes under the materials highlights an emotional and philosophical connection, where the material is not just manipulated but actively nurtured and respected. As Participant 8 continues, “Kombucha is more evolved than we are in many ways. It has been around for thousands of years and only now are we finally starting to reimagine its applications that respect its history.” This statement reflects an awareness that bacterial cellulose—derived from kombucha and alternative sources—has a long-standing existence and history of human use, yet it is only now that we are beginning to explore its potential in modern, ethical applications. There is an acknowledgement of the wisdom embedded in its evolutionary past, a reminder that its applications must be approached with reverence for the process that has sustained it over millennia.

Achieving holistic integration requires a commitment to sustainable practices that extend beyond immediate material applications and the consideration of regenerative practices. Stakeholders embracing this perspective often emphasize responsible stewardship, recognizing their role as custodians of bacterial cellulose-producing strains and material. This stewardship entails adopting practices that safeguard the well-being of these living entities, acknowledging their interconnectedness with the environment. Stakeholders advocate for conscious decision-making, considering the broader ecological impact of material extraction and production processes. This approach aligns with a larger movement towards ethical biomaterial use, emphasizing the need for responsible and mindful utilization.

### Did you say ‘bacterial’ cellulose? Ew!

This subsection discusses the public perception challenges, and the strategies needed to foster broader acceptance and understanding of bacterial cellulose’s role as a living biomaterial. Several participants experienced challenging perceptions in explaining their work with bacterial cellulose to different audiences, consumers, or demographics.

Participant 17, lecturer and creative practitioner in Australia describes the visceral reactions they face when discussing bacterial cellulose, “If you say you are working, playing, and touching bacterial cellulose, [their face] is one of horror. And then you say actually, no, it is good probiotics. You eat yogurt and drink kombucha.” Similarly, Participant 13, a textile scientist based in the United Kingdom recounts how their department reacted to the early stages of their bacterial cellulose research:

“Initially, we had to present our work to groups in our department. We said, we’re growing fabric from bacteria, and they all went, oh God, that’s horrible. That’s really horrible! We were laughing and I said, but there are more bacteria on your skin and on the clothing that you are wearing now than there is on this piece of material that I’m showing you.” (Participant 13).

Despite the fact that our bodies are home to trillions of bacteria^41^, these initial reactions highlight a significant barrier to the commercialization of BC, particularly in business-to-consumer industries. Many consumers are unfamiliar with what a biomaterial created from bacteria might look like and often assume that it is ‘living’ and harboring bacteria on its surface^42^. However, bacterial cellulose is typically purified during production to remove any remaining cells or microbes, ensuring its safety for use in products^43^.

In response to these challenges, participants adapted their messaging strategies to suit different demographics. For example, Participant 17, Lecturer and creative practitioner in Australia notes that children, with their natural curiosity were more open to the material: “Kids are great because they are not risk adverse. They are kind of going, oh my God, it is gooey. This is fantastic!” For adult audiences, particularly those outside of academia, the messaging was adjusted to focus on the material’s familiarity and health benefits. As Participant 17 continues, “[For] adults, [the story is, this is a] Kombucha health drink. We can do this.” By relating bacterial cellulose to familiar, everyday products like kombucha, participants were able to ease people into the idea of microbes as creators of materials. This approach emphasized the material’s sustainability, safety, and the growing interest in biomaterials, making it more approachable and less intimidating. The use of clear, engaging storytelling was crucial in demystifying bacterial cellulose and positioning it as both a sustainable and safe alternative to traditional materials.

The power of media in shaping public perception was evident in a June 2024 segment on *The One Show*, which featured bacterial cellulose leather^44^. During the segment, the host asked passersby if they would wear bacteria, and most responded negatively, except for one individual familiar with sustainable materials. This negative reaction may stem partly from being asked the wrong question by the host: the material is produced by bacteria rather than being ‘bacteria’. Interestingly, one of the scientists featured in the segment referred to the material as ‘nanocellulose’ instead of ‘bacterial cellulose’, reflecting a shift in terminology within the bacterial cellulose research community. Over the years, terms like bacterial nanocellulose and microbial nanocellulose have evolved due to perceptions and scientific discussions, leading to the adoption of names like ‘biocellulose,’ as used by companies such as JeNaCell. This evolution in nomenclature underscores the need to address public perceptions and improve understanding of bacterial cellulose as a sustainable biomaterial.

### Bacterial cellulose’s potential as a programmable material

Bacterial cellulose stands at the forefront of a transformative era in various disciplines such as material science, biotechnology, and synthetic biology, particularly within the realm of engineered living materials. Its fundamental simplicity as a pure cellulose producer positions it uniquely for advanced modifications. Participant 11, Professor in synthetic biology in the United Kingdom encapsulated this potential, succinctly puts it, “We aim to write DNA programs into cells to create materials with predefined functions. Bacterial cellulose is an ideal starting point for this: the cells produce plain linear cellulose in high volumes.” The programmability of bacterial cellulose and its microbe producers extends beyond mere material alteration; it represents a paradigm shift in material creation. Bacterial cellulose can be functionalised with novel properties – from enhanced mechanical strength to unprecedented biocompatibility. As Participant 18, postdoctoral research associate in synthetic biology in Australia expounds, **“**You can attach a small tag to proteins that specifically target and bind strongly to cellulose. This allows for the addition of enzymes, proteins, and other elements onto the SCOBY, thereby endowing the SCOBY with new functionalities.**”** This capacity transforms bacterial cellulose to an active participant in a range of applications, paving the way for breakthroughs in diverse fields, including sustainable textiles, biomedical devices, and environmentally responsive architectures.

Beyond genetic reprogramming of the microbial cells, bacterial cellulose can also be “programmed” through other methods. These include adapting the cultivation conditions^8^, adding specific additives^2^, co-culturing the bacterial cellulose strains with other microorganisms such as *Saccharomyces cerevisiae*^27^, and biofabrication^45^. Moreover, there are opportunities for pre-cultivation and post-cultivation modifications. Pre-cultivation adjustments can include altering the nutrient medium^8^, while post-cultivation modifications might involve chemical treatments to enhance or add new functionalities to the bacterial cellulose such as stimuli-responsive release mechanisms which allow the attached moieties to perform specific functions in response to environmental triggers^3^. These diverse approaches to programming bacterial cellulose highlight its versatility and potential for innovation across numerous applications.

Delving into the processes of working with programmable materials like bacterial cellulose reveals an intersection of science and design. Participants all over the human-living material relationship spectrum and regardless of discipline expressed interest in the development and modification of bacterial cellulose. As Participant 14, Chief Scientific Officer of a material company in Denmark shares, “The journey with bacterial cellulose challenges traditional material boundaries. It’s about harnessing its inherent properties and reimagining them through a biotechnological lens.” This innovative process is not linear but rather iterative and exploratory, requiring both scientific and design elements. Scientists and designers engage in a dialogue with the material and microbes, questioning established norms and venturing into uncharted territories. Participant 5 a creative arts practitioner in Austria, elaborates:

“I would recommend delving into existing scientific knowledge as a foundation but also challenging and expanding beyond it. Our project’s most pivotal advancements occurred when we ventured into uncharted territory, experimenting with ideas that had no guaranteed potential or precedent. It was about taking the leap and trying something new, even when no one could predict its outcome.”

The design process with bacterial cellulose is marked by a unique set of considerations compared to other design processes – understanding the genetic makeup of the cellulose-producing bacteria, the environmental factors influencing production, and the downstream implications of material modifications. Participant 19, CEO of a material startup focusing on developing bacterial cellulose in Hungary notes, “Working with living organisms is both an advantage and disadvantage at the same time. The possibilities of genetic modifications to the microbes or material modifications to the cellulose are endless.” There is a myriad of ways that programmability and modifications with bacterial cellulose or its strains that produce it can be programmed. In future research, a more structured understanding of these possibilities will be crucial in advancing the programmability of bacterial cellulose and its integration into mainstream applications. Ultimately, it will require sustained collaboration across disciplines to realize the full potential of bacterial cellulose as a truly programmable, multifunctional material.

## Discussion

**Fig. 2:**
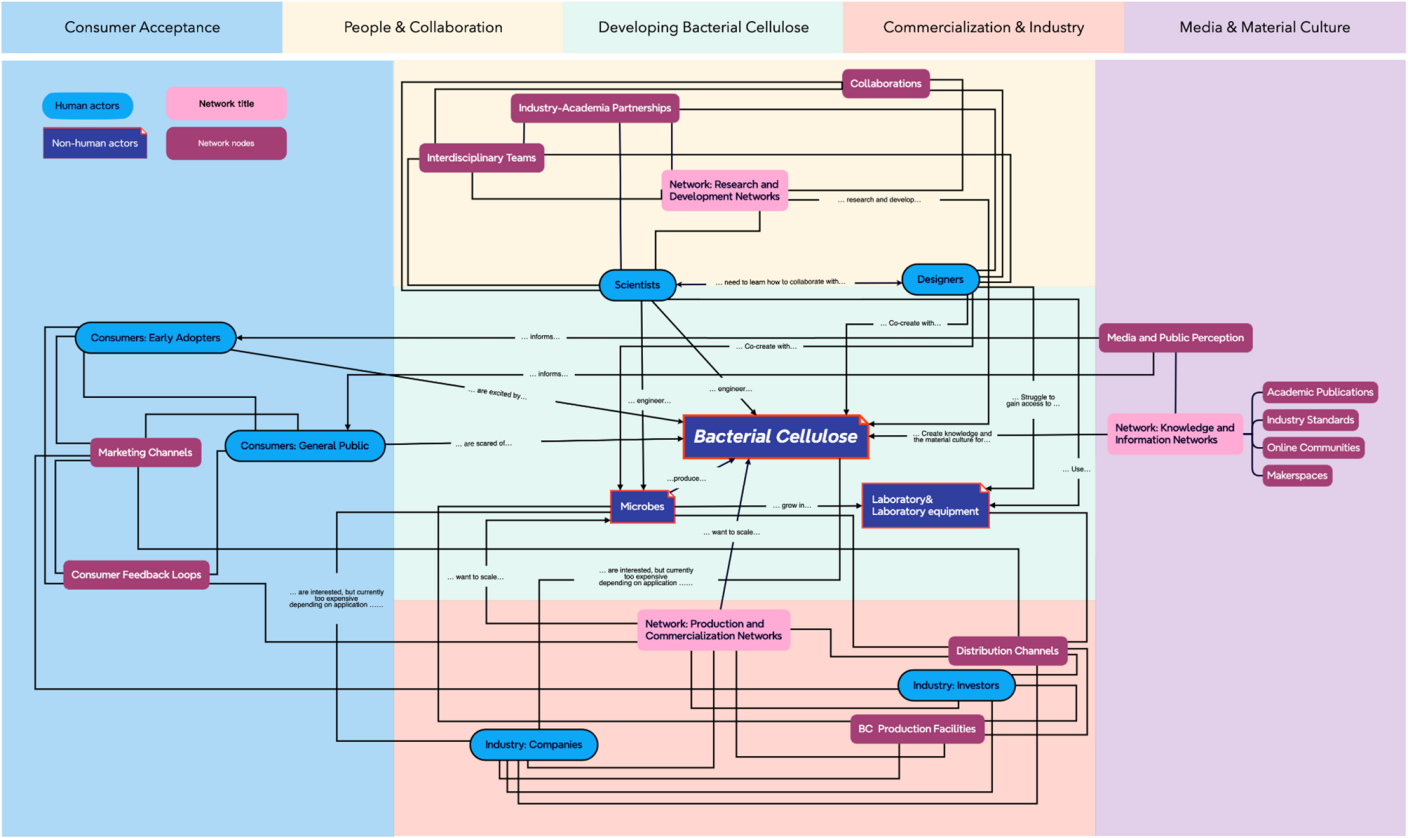
Actor-Network Theory visual network of bacterial cellulose, illustrating the interconnected relationships between human and non-human actors. The network map highlights key clusters including consumer acceptance, people & collaboration, developing bacterial cellulose, commercialization & industry, and media & material culture, showing how these elements influence and shape the development, perception, and future trajectory of bacterial cellulose as an engineered living material.

The results of this study highlight the intricate network of human and non-human actors involved in the development and application of bacterial cellulose as an engineered living material and can be seen in Fig. 2 on the next page. By employing Actor-Network Theory—a framework developed by Bruno Latour that explores how both human and non-human entities, such as bacteria, materials, and technologies, interact and influence one another within a network^46^ — we gain a nuanced understanding of the dynamic relationships that shape bacterial cellulose’s evolution and acceptance across various industries. As a single component within this broader system, bacterial cellulose’s trajectory is influenced by myriad other actors and forces, often in ways not immediately perceptible. The network map presented in this study serves as a starting point, reflecting the primary author’s interdisciplinary perspective from design and science, and aims to stimulate further exploration into bacterial cellulose’s role as a biomaterial.

### Consumer acceptance

Consumer acceptance is a critical factor in determining the success of any new material, particularly one as novel as bacterial cellulose. Our findings indicate that consumer attitudes toward bacterial cellulose are heavily shaped by presentation strategies and the narratives constructed around its use. Many participants in both design and science emphasized the importance of crafting compelling material stores that align with consumer values and address potential concerns, especially for business-to-consumer industries. A recurring theme was the challenge of overcoming the “ew factor” associated with bacterial cellulose’s microbial origins. Consumers unfamiliar with biomaterials may perceive bacterial cellulose as unsanitary or unappealing, despite its biocompatibility, sustainability, and purification processes.

As a result, designers and marketing professionals should consider reframing bacterial cellulose not just as a material but as an eco-conscious innovation that aligns with growing consumer demand for sustainable products. One effective approach for fostering consumer acceptance involves associating bacterial cellulose with familiar products, such as kombucha or probiotic foods, which already enjoy widespread consumer trust. Future studies should explore the impact of long-term consumer engagement strategies, material literacy campaigns and educational initiatives, in-store demonstrations, and sustainability certifications, to enhance bacterial cellulose’s acceptance in various industries. Addressing consumer hesitancy is not just a marketing challenge but a necessary step in the material’s integration into mainstream commerce.

### People & collaboration

Collaboration between stakeholders is essential for the successful development and commercialization of bacterial cellulose. By applying Actor-Network theory, we can see how human actors (designers, scientists, industry professionals) and non-human actors (the material itself, technology, institutional frameworks) interact in a network that shapes the trajectory of bacterial cellulose^46^. Our findings reveal that collaboration is most productive when stakeholders develop mutual respect for differing methodologies, objectives, and disciplinary perspectives. For instance, scientists and designers engage with bacterial cellulose differently, yet their approaches are complementary. Scientists prioritize material functionality, focusing on consistency, performance, and industrial scalability. Their work involves optimizing bacterial cellulose production, ensuring reproducibility, and developing standardized processing methods that make bacterial cellulose commercially viable. In contrast, designers approach bacterial cellulose with a speculative, iterative lens, exploring aesthetic, tactile, and conceptual possibilities. Their focus lies in prototyping, challenging material paradigms, and envisioning speculative futures for bacterial cellulose.

Although both disciplines contribute to bacterial cellulose’s advancement, their collaboration is often asymmetrical. A rising number of designers are embedding themselves in scientific research and processes, working in laboratory environments to refine bacterial cellulose’s properties^47^. This integration broadens research perspectives, introduces novel problem-solving approaches, and encourages exploratory methodologies that might not otherwise be pursued in scientific settings. However, there is limited research of scientists engaging directly in design studios to explore bacterial cellulose’s creative and experiential potential. This asymmetry presents an opportunity for deeper cross-disciplinary exchanges. By embedding scientists in design-led experimentation, research teams can uncover new applications, cultural narratives, and interactive possibilities for bacterial cellulose. To facilitate these exchanges, institutions and companies could establish shared workshops, hybrid research teams, or interdisciplinary residencies, where designers and scientists work collaboratively rather than in parallel. Strengthening these connections will be critical in unlocking new applications for bacterial cellulose across scientific, industrial, and creative sectors.

### Developing bacterial cellulose

Bacterial cellulose development extends beyond technical optimization and involves a complex interplay of actors, industries, and societal influences. While laboratory research focuses on enhancing bacterial cellulose’s physical and functional properties, this study’s thematic analysis and Actor-Network theory analysis reveal that bacterial cellulose is shaped by a broader ecosystem, including media representation, global research initiatives, and material culture. A crucial insight from our findings is that the future of bacterial cellulose does not rest solely on scientific advancements but also on how stakeholders collaborate, communicate, and integrate the material into mainstream discourse. This means that bacterial cellulose’s long-term success as a biomaterial depends on engaging public perception, regulatory acceptance, and industrial adaptability.

### Commercialization & industry

Commercializing bacterial cellulose requires navigating a complex landscape of market dynamics, regulatory approval, and industrial adoption. The participants in our study, particularly those from industry backgrounds, emphasized the importance of establishing clear value propositions that attract investment and demonstrate bacterial celluose’s versatility. This includes highlighting its sustainability credentials, customizability, and potential for high-performance applications. Despite the proven scalability of bacterial cellulose production, the primary commercial challenge is not capacity but material adaptation. Companies such as JeNaCell (Germany), Cellulose Lab (Canada), and Polybion (Mexico) have successfully scaled bacterial cellulose in different formats such as fibers, fleeces, and slurry-based applications. Yet, its industrial adoption remains limited by cost, modification and regulatory complexities.

Key commercialization hurdles include: (1) optimizing bacterial cellulose for specific industries (textiles, biomedical applications, packaging, etc.); (2) developing cost-effective processing techniques that enhance durability and performance; (3) navigating regulatory standards that dictate bacterial cellulose’s use in consumer applications; (4) technology transfer between research institutions and industry partners to streamline adoption. Future industrial adoption of bacterial cellulose will require strategic partnerships between academia, business, and government regulators. These collaborations will help standardize processing techniques, certify applications, and expand bacterial cellulose’s presence in global markets.

### Media & material culture

Media and cultural narratives play a significant role in shaping public perceptions of bacterial cellulose and, by extension, its acceptance and use. The participants in our study noted that the way bacterial cellulose is represented in media—whether through scientific publications, marketing campaigns, or popular culture—can significantly influence its material culture. For example, media coverage that highlights bacterial cellulose’s role in sustainability can help position it as a desirable material for eco-conscious consumers. This cluster emphasizes the need for strategic communication efforts that can help build a positive material culture around bacterial cellulose, facilitating its integration into society.

### Future areas of research and limitations

This study provides valuable insights into the role of scientists and designers in the development of bacterial cellulose. However, it is important to recognize a few limitations that can be addressed in future research. First, while the study focused on scientists and designers, it did not include other key stakeholders, such as policymakers and consumers, who play significant roles in the adoption and application of bacterial cellulose. Including these perspectives would offer a more comprehensive understanding of how the material is framed across different sectors. Second, another limitation of this study is the use of Zoom for interviews. While this method allowed us to engage with participants from 11 countries, it may have constrained the depth of interaction compared to in-person interviews. Non-verbal cues and the opportunity for more spontaneous exchanges may have been missed. Future research could benefit from a hybrid approach, combining remote interviews with face-to-face sessions.

Lastly, while the study primarily addressed technical aspects and material development, it did not dive deeper into the ethical implications of bacterial cellulose’s use. Future studies could explore the human-living material relationship spectrum through ethical frameworks, such as utilitarianism, and incorporate perspectives from Social and Human Sciences (SHS) to assess whether bacterial cellulose’s applications align with broader societal goals like sustainability and reduced consumption. Jeremy Bentham’s principle of utility suggests that actions are ethically sound when they maximize overall happiness or welfare^48^. However, in practice, advanced technologies may often prioritize cost-efficiency over long-term well-being and sustainability. Future research should more explicitly explore how the decisions of scientists, designers, and policymakers may prioritize certain applications over others based on perceived utility rather than holistic ethical considerations, and how SHS-based frameworks can help uncover and challenge these value hierarchies. Incorporating ethical reflexivity and interdisciplinary critique will be essential for evaluating not only how materials like bacterial cellulose are developed, but also why and for whom they are deployed.

## Conclusion

This study is the first to systematically compare how scientists and designers from many disciplines interact and develop bacterial cellulose, revealing distinct disciplinary perspectives. Scientists primarily focus on bacterial cellulose’s functionalization, efficiency, industrial scalability, and optimizing its properties for specific applications. In contrast, designers typically engage with bacterial cellulose through an iterative and speculative lens, emphasizing sensory experience, material storytelling, and its potential to challenge conventional paradigms. The human-living material relationship spectrum—from utilitarian viewpoints to holistic integration—illustrates the diverse relationships stakeholders develop with bacterial cellulose as both a material and a living entity. Perspectives on bacterial cellulose’s programmability further highlight its potential for dynamic material innovation. Actor-Network theory contextualizes these relationships, illustrating the network of human and non-human actors shaping bacterial cellulose’s adoption. As bacterial cellulose continues to evolve, its future will be defined not by a single discipline but through the convergence of science, design, and industry—where collaboration, creativity, and critical inquiry will drive its most transformative applications.

## Data Availability

The original contributions presented in the study are included in the article and the data utilized in this study is subject to confidentiality restrictions. Further inquiries are encouraged to contact the corresponding author.
